# Wet Pack in Scarlet Fever

**Published:** 1884-02

**Authors:** 


### Wet Pack in Scarlet Fever.

Dr. A. F. Rundall, in *Medical Call,* speaks highly of this remedy. He says he always puts the patient in a wet pack, if called before the rash is thoroughly out, and before desquamation begins to take place, using water at the temperature of 67 to 70 degrees. The patient is left in the pack from half to three-quarters of an hour, and is given all the cold water he wants to drink. The pack may be repeated to reduce fever. He finds this treatment to act nicely in allaying fever and bringing out the rash.—*Md. Med. Jour.*

